# Current status of implant prosthetics in Japan: a survey among certified dental lab technicians

**DOI:** 10.1186/s40729-015-0005-3

**Published:** 2015-02-17

**Authors:** Yoshiyuki Hagiwara, Tatsuya Narita, Yohei Shioda, Keisuke Iwasaki, Takayuki Ikeda, Shunsuke Namaki, Thomas J Salinas

**Affiliations:** 1 Implant Dentistry, Nihon University School of Dentistry, Dental Hospital, 1-8-13 Kandasurugadai, Chiyoda-ku, Tokyo 101-8310 Japan; 2 Department of Dental Specialties, Mayo Clinic, Mayo Clinic, 200 First Street SW, Rochester, MINN 55905 USA

**Keywords:** Questionnaire, Survey, Implant prosthesis, Certified dental technicians, Prosthetic complications

## Abstract

**Background:**

There are many implant cases in which dental technicians take initiative with regard to the design of implant prostheses, and to a certain extent, this area of care is one in which dentists do not necessarily play the leading role. Moreover, inadequate communication between dental technicians and dentists and insufficient instructions for technicians has been highlighted as issues in the past. The purpose of this questionnaire is to improve the quality of implant prostheses and thereby contribute to patient service by clarifying, among other aspects of treatment, problem areas and considerations in the fabrication of implant prostheses, conceptual-level knowledge, and awareness of prosthodontics on the part of the dentists in charge of treatment and methods for preventing prosthetic complications.

**Methods:**

A cross-sectional survey was given to 120 certified dental technicians. To facilitate coverage of a broad range of topics, we classified the survey content into the following four categories and included detailed questions for (1) the conditions under which implant technicians work, (2) implant fixed prostheses, (3) implant overdentures, and (4) prosthetic complications.

**Results:**

Out of 120 surveys sent, 74 technicians responded resulting in a response rate of 61.6%.

**Conclusions:**

This survey served to clarify the current state of implant prosthodontics, issues, and considerations in the fabrication of implant prostheses, and the state of prosthetic complications and preventive initiatives, all from a laboratory perspective. The results of this survey suggested that, to fabricate prostheses with a high level of predictability, functional utility, and aesthetic satisfaction, it is necessary to reaffirm the importance for dentists to increase their prosthetic knowledge and work together with dental technicians to develop comprehensive treatment plans, implement an organized approach to prosthesis design, and accomplish occlusal reconstruction.

## Background

Currently, dental implant treatment is evaluated on the basis not only of restoring masticatory function, but also a variety of other factors, including the implant and superstructure survival rate and psychological impacts [1-3]. Numerous factors must be taken into account, to offer highly predictable implant treatment, and there is no doubt that prosthetic-related factors such as the type and compatibility of the prosthesis, as well as occlusion, make a major contribution to that goal [4-9].

Recently, a restoration-driven approach to implant treatment has gained recognition and is being put into practice on a broad basis [10,11]. However, an increasingly diverse range of patient cases has led to a situation in which it is impossible to ascertain such aspects of actual practice as prosthesis type and design, making it necessary to reaffirm the importance of treatment carried out from a prosthetic perspective [12]. Many surveys querying dentists or patients with regard to implant treatment have been reported in the literature, addressing such topics as the state of implant treatment in particular countries and regions [13,14], quality of life and patient satisfaction [15-17], peri-implantitis and mucositis [18], and implant education [19,20]. However, very few surveys have queried dental technicians, whose job it is to fabricate implant prostheses [21,22].

Dental technicians play a major role in current implant treatment because of increases in both the importance of their participation as part of the treatment team from the treatment planning stage [21] and the frequency of prosthesis repairs, refabrication, and related procedures in the event of prosthetic complications. In particular, the types of prosthetic complications being experienced and associated trends are becoming clear thanks to numerous systematic reviews undertaken recently to investigate the implant complications. Fixed prostheses are prone to issues such as screw loosening, crown detachment, and fracturing of the veneering material on a frequent basis [23-27]. Similarly, implant overdentures are frequently affected by progressive loosening of attachments, denture base fractures, and a sequential need for relining [28,29]. However, because understanding the status of these complications is based on the results of surveys targeting dentists, information is needed on the situation as seen from the standpoint of implant technicians, to clarify the causes of these complications and the techniques for dealing with them. Issues including inadequate communication between dental technicians and dentists and insufficient instructions for technicians have been pointed out in the past [21,30,31]. These reports derive from surveys targeting older fixed or removable prosthesis designs, leaving it unclear not only whether those issues have been rectified in the face of expanding use of implant prostheses in recent years, but also to what degree the opinions and wishes of dental technicians are being reflected in implant treatment.

This survey consists of a questionnaire targeting the certified dental technicians of the Japanese Society of Oral Implantology (JSOI) [32] who are primarily involved in fabricating dental implant restorations. It was formulated to clarify the current status of implant prostheses from a prosthetic and technician-oriented standpoint through questions addressing current trends among dental implant technicians, fixed prostheses, implant overdentures, and prosthetic complications and measures. The certified dental technicians of JSOI queried by the survey are involved in implant-related laboratory work on a comparatively frequent basis, and the responses they provided can be expected to accurately reflect the current state of implant laboratory practice in Japan. Our goal through this questionnaire is ultimately to improve the quality of implant prostheses and thereby contribute to patient service. We aim to do this by clarifying, among other aspects of treatment, problem areas, and considerations in the fabrication of implant prostheses, the conceptual-level knowledge base and awareness of prosthodontics on the part of the dentists in charge of treatment and methods for preventing prosthetic complications.

## Methods

This cross-sectional questionnaire survey was performed among the certified dental technicians of JSOI from September to December in 2011. Selected were 120 out of 285 certified dental technicians of JSOI using a random number table and mailing each questionnaire directly to the participant. To facilitate coverage of a broad range of topics, the survey classified content into the following four categories and included detailed questions for each: (1) the conditions under which implant technicians work (questions 1 and 2); (2) implant fixed prostheses (methods of retention, abutment, and prosthesis types; questions 3–6); (3) implant overdentures (questions 7 and 8); and (4) prosthetic complications (complication types, methods of treatment and prevention; questions 9–14). Details of the questions and results are provided in Tables 1, 2, 3, and 4. Given that no previous survey regarding implant dental technician data had been developed, an original form for this purpose was constructed following suggested guidelines [33,34]. Important to the construction validity, both the questionnaire authors and their audience were clinical specialists and were aware of the topic content. The content sought in the questionnaire was a measure of responder demographics, clinical experiences, and subjective perceptions. Additionally, interpretation errors were minimized because of content familiarity and standardization, which improved reliability, and no pretest measures were obtained given the mail-based assessment method.

**Table 1:** **Conditions characterizing implant laboratories**

**Question**		**Values**
Q1. The years of experience working as a dental technician, and the number of dentists from whom job orders are received.		Mean (SD)
		17.0 (6.8) years
		36.5(12.4)/Lab.
Q2. Who takes the leading role in treatment planning and prosthetic design (initiative with regard to prostheses)?	Dentists mainly exercise initiative	39.3%
	Technicians mainly exercise initiative	15.0%
	Technicians are often consulted when it comes to specific cases and parts	16.8%
	Decisions are made in collaboration with each other	28.9%

**Table 2:** **Implant fixed prostheses**

**Question**		**Values**
Q3. The percentages of implant fixed prostheses:	Cement-retained	61.4%
	Screw-retained	38.6%
Q4. What are the proportions of abutments used with cement-retained prostheses?	CAD/CAM (titanium)	19.7%
	CAD/CAM (zirconia)	12.1%
	Custom abutments (UCLA-type abutment + gold alloy)	33.2%
	Two-piece-type titanium (prepable type)	28.3%
	Other	6.5%
Q5. What types of materials (i.e. veneer, coping) are used to make implant prostheses in the anterior region?	Porcelain fused to metal crown	43.4%
	All ceramic crown (zirconia)	27.1%
	All ceramic crown (other materials)	6.6%
	Indirect composites (facing crown)	21.3%
	Indirect composites (jacket crown)	2.4%
Q6. What types of implant fixed prostheses are used in the posterior region?	Porcelain fused to metal crown (full bake)	31.4%
	Porcelain fused to metal crown (metal occlusal)	9.1%
	All ceramic crown (zirconia)	14.3%
	Indirect composite veneer crown (full bake)	22.3%
	Indirect composite veneer crown (metal occlusal)	12.6%
	Metal crown	10.3%

**Table 3:** **Implant overdentures (IODs)**

**Question**		**Values**
Q7. The design of the implant overdenture:	Decision made according to instructions of dentist	43.2%
	Work is left to technicians	19.3%
	Decided upon through consultation with each other	37.5%
Q8. What are the proportions of attachment types used with IODs?	Bar and clip	35.6%
	Magnet	30.2%
	Ball and socket	19.0%
	Locator	5.2%
	ERA	2.3%
	Other	7.7%

**Table 4:** **Prosthetic complications**

**Question**		**Values**
Q9. What are the main issues generally encountered?	Compatibility precision issues	29.6%
	Aesthetic issues	33.2%
	Occlusal issues	37.2%
Q10. What are the main fabrication challenges faced?	Poor implant location and orientation	42.4%
	Inadequate consideration of occlusion	17.0%
	Defects and inaccuracies in impression and bite registration	29.0%
	Defective or unreasonable prosthesis design	10.6%
	Other	1.0%
Q11. What are the frequently received repair requests involving implant fixed prostheses?	Facing damage and chipping	54.5%
	Facing discoloration and wear (indirect composite veneer crowns)	17.0%
	Bridge connector fracture	10.0%
	Design changes and modification associated with additional implants	13.9%
	Other	4.6%
Q12. What kind of creative steps do you take in order to prevent veneer fracture and chipping in the molar region?	Use of metal occlusal designs	15.1%
	Use of indirect composite resin material	15.7%
	Devise metal coping designs	36.3%
	Cover the distal-most part with metal	24.0%
	Nothing in particular	8.9%
Q13. What are the frequently received repair requests for IODs?	Fracturing of the denture base or denture tooth detachment/fracture	53.8%
	Mesostructure (attachment) damage	8.4%
	Occlusal reconstruction due to denture wear or attrition	24.1%
	Replacement of the attachment system (transition to another system)	8.1%
	Other	5.6%
Q14. Do you have any requests for dentists who practice implant treatment?	To consult technicians or allow technicians to participate from the treatment planning stage	28.3%
	To use suitable implant location and orientation	31.8%
	To improve treatment and condition of soft tissue	21.8%
	To study more about prostheses and occlusion	14.5%
	Other	3.6%

## Results and discussion

Out of 120 surveys sent, 74 technicians responded, resulting in a response rate of 61.6%. A summary of the responses is provided in Tables 1, 2, 3, and 4 and Figures 1, 2, 3, 4, 5, 6, 7, 8, and 9.

**Figure 1:**
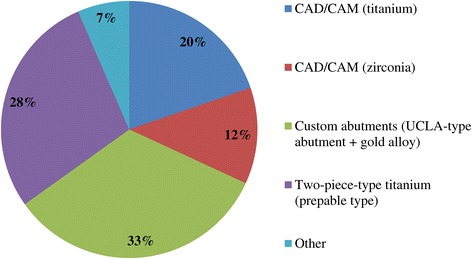
**Q4. What are the proportions of abutments used with cement-retained prostheses?**

**Figure 2:**
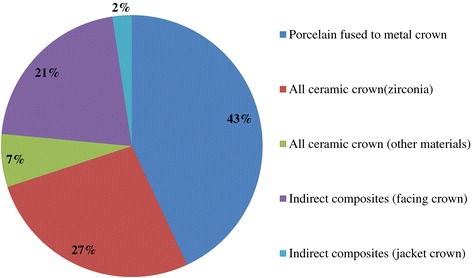
**Q5. What types of materials (i.e. veneer, coping) are used to make implant prostheses in the anterior region?**

**Figure 3:**
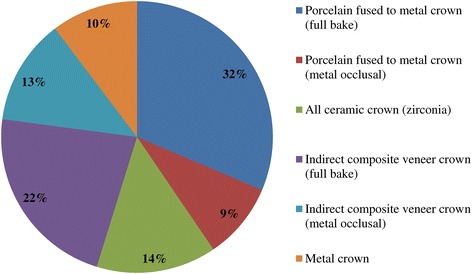
**Q6. What types of implant fixed prostheses are used in the posterior region?**

**Figure 4:**
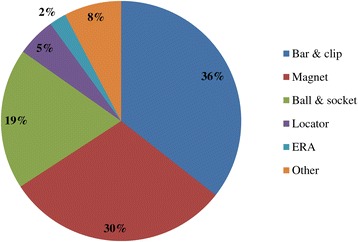
**Q8. What are the proportions of attachment types used with IODs?**

**Figure 5:**
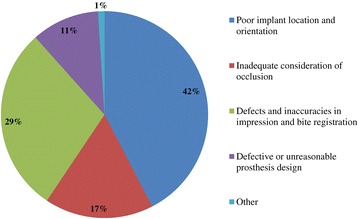
**Q10. What are the main fabrication challenges faced?**

**Figure 6:**
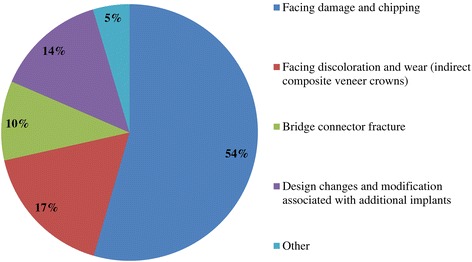
**Q11. What are the frequently received repair requests involving implant fixed prostheses?**

**Figure 7:**
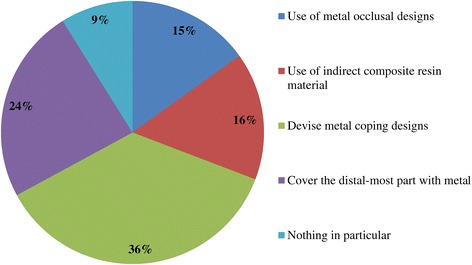
**Q12. What kind of creative steps do you take in order to prevent veneer fracture and chipping in the molar region?**

**Figure 8:**
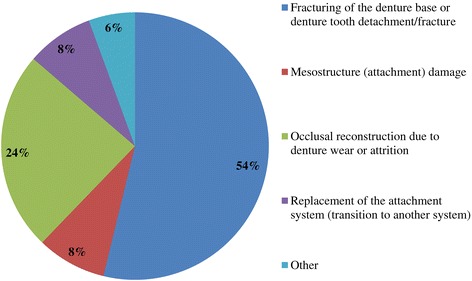
**Q13. What are the frequently received repair requests for IODs?**

**Figure 9:**
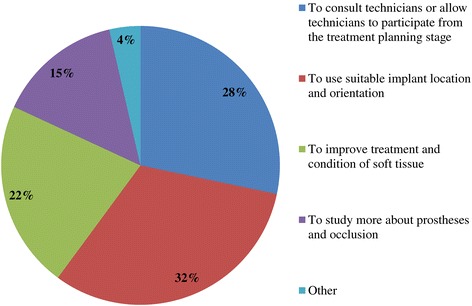
**Q14. Do you have any requests for dentists who practice implant treatment?**

Because implant treatment (implant prostheses) requires a significant amount of specialized, high-precision laboratory procedures, this area of dental care exhibits slightly different trends than prosthetic treatment as it was practiced in the past, and this work is concentrated at specialized fabrication labs. Moreover, there are many cases in which dental technicians take initiative with regard to the design of implant prostheses, and to a certain extent, this area of care is one in which dentists do not necessarily play the leading role. In light of these circumstances, it was intended for this questionnaire to verify trends in implant treatment from a different perspective than has been used in the past, by investigating the current state of practice in the field from the dental technician perspective. By evaluating implant treatment from the standpoint of dental technology/prosthodontics and identifying current trends and problem areas, it was expected to gain information that enables highly predictable implant treatment.

1. Conditions characterizing implant laboratories (Table 1)
  The dental technicians who responded to this questionnaire have an average of about 17 years of experience in the field, indicating that they possess an adequate level of fabrication experience. In light of the reality that dental implant treatment is a comparatively new field, these personnel can be proficient with digital techniques as they differ from past generations of technicians who practiced the craft. On average, each dental technician serves about 36.5 customers, although that number varies depending on the scale of the fabrication lab at which they work. While implant laboratory work consists of complex processes, the fees are high, and labs generate a stable flow of revenue given a constant stream of work requests (Q1).
  Dentists play a leading role in 39.3% of the time in implant treatment planning and prosthetic design, and dental technicians are consulted concerning cases and part usage 34.7% of the time, suggesting the approach to implants is driven by prosthetic considerations (by dentists) to some degree. However, because dental technicians indicated that they take the initiative 15% of the time, it is impossible to ignore issues involving the care, skill, and judgment of dentists offering implant treatment. This is distinct from the question of whether communication or information transmission between dentists and dental technicians is adequate, but rather relates to implant treatment knowledge, especially decisions about which prostheses and other treatment tools to use. The repercussions of this problem extend to the rate of incidence of prosthetic complications occurring after the start of functional use, their prevention, and the measures that are undertaken to address them (Q2).
  Education of dental technicians varies by country, and there are a variety of means by which personnel master fabrication knowledge and skills. For example, a survey of dental technicians in the UK conducted by Bower et al. [35] reveals that while subjects read commercial magazines published for dental technicians, they rarely subscribed to academic journals in the field of prosthodontics, and two thirds of the survey’s respondents had never attended a training course on fabrication practices. By contrast, certified dental technicians of JSOI are required to belong to an academic society and to participate in society meetings and certification courses to maintain their credentials. Subscription to JSOI’s journal is an example of the advantages of membership for continuing education.
2. Implant fixed prostheses (Table 2)
  Implant fixed prostheses employ either cement or screw retention. While there are a variety of reports comparing the two methods in terms of such metrics as their respective prognoses, success rates, and advantages and disadvantages [7,36-38], no reports have been published concerning their relative frequency of use. Our questionnaire indicated a distribution of 61.4% cement-retained versus 38.6% screw-retained prostheses (Q3), suggesting that cement retention is used more frequently in Japan. Unfortunately, the fabrication-oriented focus of this survey prevented clarification of the types of cement used for cement retention and the breakdown between provisional and definitive cement.
  Next, concerning the types of abutments used with cement-retained prostheses (Q4) (Figure 1), CAD/CAM abutments accounted for about one third of the total (titanium, 19.7%; zirconia, 12.1%), and custom UCLA-type abutments made from cast gold alloy accounted for about the same proportion. It is likely that this breakdown is because, in many cases, implant systems using fabricated crowns are not supported by CAD/CAM abutments. CAD/CAM system use is also subject to numerous limitations because of the licensing process imposed by the Ministry of Health, Labour and Welfare (MHLW) in Japan, which is strict when compared with its constituents in other countries. The questionnaire also indicated that titanium two-piece abutments (preparable type) are used in about the same proportion; 28% of the time. This reflects such factors as efforts to keep laboratory costs down and to shorten delivery time frames, in addition to the above reasons.
  Concerning the types of prostheses used in the anterior region (i.e., veneering materials), the questionnaire indicated a trend toward selection of roughly the same materials for both single crowns and bridges (Q5) (Figure 2). As a rule, porcelain fused to metal (PFM) crowns accounted for 43.7% of the total, but selection of metal-free restorations using zirconia has been increasing in recent years, reaching approximately 27.1%. Incidentally, veneering porcelain was also used as the veneer material for zirconia copings. The questionnaire also indicated that while highly filled indirect composites such as Estenia (Kuraray, Osaka, Japan) were used 21.3% of the time, primarily for facing crowns, these materials were used infrequently for jacket crowns (2.4%). There is a low risk of facing damage and chipping for prostheses in the anterior region. Nonetheless, the questionnaire revealed the unexpected result that indirect composite facing crowns accounted for 21.3% of the total. This may be because there are many indirect composite resins (Estenia, Ceramage, etc.) available in Japan, and crowns and bridges in the anterior region (natural abutment teeth) are covered by certain types of insurance in the country (National Health Insurance and Social Insurance), with the result that Japanese dentists are familiar with these materials and use them frequently. Consequently, it can be surmised that using these materials in implant prostheses is more common than in Europe and the USA. However, no survey of prosthesis selection has yet been carried out, and future research on that subject is expected.
  Concerning the types of prostheses used in the posterior region (Q6) (Figure 3), PFM design accounts for about 40% of the total, although the questionnaire also revealed a trend (in 9.1% of all cases) toward metal occlusal designs to avoid fracture and chipping of the veneer material. The same trend is evident in indirect composite facing crowns, where metal occlusal designs are used in about 35% of all cases that this type of prosthesis represents. In the past, the PFM crown was frequently used in implant crowns and bridges. However, a trend is seen toward increasing indirect composite resin use as a veneer material for implant superstructures. In addition to improvements in the physical properties (strength, wear resistance, and discoloration resistance) of indirect composites in recent years, their selection as veneer materials that chemically bond to titanium against the backdrop of increasing CAD/CAM-designed titanium frameworks, because of the low reliability of veneering porcelain, in terms of bonding strength, when used with titanium frames. There is also a greater possibility of direct (in-mouth) repair of failed veneering materials and greater shock-absorbing potential relative to occlusal force in comparison with porcelain [39]. The trend to adhere resin materials instead of porcelain, from Brånemark and colleagues’ recommendations for acrylic resin as an occlusal surface material in the early 1980s, also cannot be ignored [40]. All metal crowns were used about 10.3% of the time in molar regions because of a lack of strong aesthetic requirements. Zirconia, however, accounted for 14.3%; only about half of its use in the anterior region. Possible reasons include this region not being an aesthetic area and veneer material fracture and chipping problems that have yet to be completely resolved [23,41,42].
3. Implant overdentures (IODs) (Table 3)
  Some 19% of IOD design work is left to technicians, while 80% is performed according to the instructions of, or in consultation with, dentists (Q7). As was the case with the question concerning overall prosthesis design described above, these results indicate that a team approach is being put into practice.
  Bar and clip attachments were most commonly used for IODs, followed by magnet, ball, and socket, and Locator attachments (Q8) (Figure 4). It is noteworthy among the questionnaire results that magnetic attachment use is highest in Asian countries, including Japan [43]. Additionally, it is thought that the low use of Locators (5.2%) is strongly influenced by Japan’s strict pharmaceutical regulations and because the MHLW in Japan had not yet licensed the device at the time the questionnaire was administered. Conversely, ball and socket attachments have been standardized by major implant manufacturers, and the freedom with which prefabricated parts can be used has led to their comparatively broad use. IOD use in Japan is by no means widespread; a survey of IOD use in ten countries by Carlsson et al. [44] revealed that the adoption rate of these devices in Japan was just 7% for individuals with mandibular edentulism. This number was lower than in any of the other nine countries, and future changes in IOD use in Japan are a topic that remains interesting.
4. Prosthetic complications (Table 4)
  According to Papaspyridakos et al. [2], indicators such as implant level (the relationship between the implant and bone) and the state of soft tissue around the implant are the most frequently used indices of implant success, followed by the presence and status of any implant prosthetic complication. Implant prosthetic complications include materials science-related factors, biomechanical and occlusion-related factors, and aesthetic factors. A systematic review of numerous complications that have been reported recently reveals the prostheses, restoration methods, materials, and areas most susceptible to complications [2,23-29]. Additionally, the frequency of prosthesis repairs, and repair costs cannot be ignored from a medical economic standpoint [2].
  Of the problems and issues generally encountered on the laboratory side, compatibility precision, aesthetic issues, and occlusal issues each accounted for about one third of the total (Q9). When these results are examined in connection with laboratory challenges (Q10) (Figure 5), it becomes clear that technicians regard poor implant location and orientation (42.4%) as obstacles to success. Many other issues derived from factors such as dentists’ skill level and treatment planning knowledge are directly related to quality implant treatment, such as defects and inaccuracies in impression-taking and bite registration (29%), inadequate establishment of appropriate occlusal schemes (17%), and deficient or unreasonable prosthesis design (10.6%). These issues can easily give rise to a variety of prosthetic complications after initiating functional use (and may also lead to biological complications), and dentists who offer dental implant treatment should reflect on improving their techniques. In particular, unsuitable implant locations, positions, and orientations can be prevented through appropriate preoperative examination and planning based on diagnostic wax-ups and surgical templates.
  Looking at repair requests (i.e., complications) involving the superstructures of fixed implant prostheses (Q11) (Figure 6), facing damage and chipping accounted for more than half of all requests (54.5%). Generally speaking, there are many reports that indicate a high incidence of complications related to fixed prostheses involving abutment screw loosening, detachment of cement-retained crowns, and veneer (porcelain/composite resin) fracturing and damage. Because this question addressed repair of implant prostheses, we did not obtain information about complications that can be resolved in a chair-side setting. However, the high rate of requests for facing repairs makes it clear that veneer material chipping and similar issues are occurring at a high frequency [25-27].
  Although the literature includes reports indicating a greater incidence of chipping and fractures for veneering porcelain than hardened resin [45,46] and for bridges than single crowns [26,27], this questionnaire does not shed light on the relative repair rates for porcelain and composite resin, nor the types of prostheses most likely to experience these issues. In the future, it would be worthwhile to conduct follow-up surveys on the differences among veneering materials and prostheses as well as veneer material failure trends.
  Other cases requiring repair seen by technicians include facing discoloration (veneering composite resin) (17%) (Figure 7) and design changes and modification requests associated with additional implants (13.9%). Studies have pointed to issues related to degradation of materials science characteristics for veneering composites that are distinct from those associated with porcelain, including loss of glossiness because of the deterioration of the surface and discoloration, wear, and attrition due to long-term use [47]. It is interesting to note how relatively frequently repairs are performed to address these issues. It has become clear that no small number of laboratory work requests deal with these issues experienced by patients undergoing implant treatment because of changes over time in the area surrounding existing implant treatments that occasionally necessitate additional implants and superstructure design changes or modifications.
  The questionnaire revealed several creative steps, based on laboratory considerations, being taken to prevent veneer chipping and fractures, a frequent and problematic prosthetic complication (Q12) (Figure 7). Technicians were taking into account metal (including zirconia) coping designs (36.3%), covering only the distal-most part of the molar region with metal (24%), using veneering composite resin (15.7%), and using metal occlusal designs (15.1%).
  The type of coping is important in preventing veneer fractures, and it is necessary to secure adequate veneering material thickness and to consider the dispersion of stress [48]. Particularly as zirconia becomes more common, there has been a move to improve coping designs using CAD/CAM and to exercise care concerning the prevention of veneering porcelain fracture [49,50]. Responses to this survey support the idea that this concept has been gaining popularity among technicians in recent years.
  Conversely, it was not expected that 15.7% of respondents would indicate that they use composite resin to prevent veneering material fractures. Moreover, there is no evidence that veneering composites are more resistant to fracture than porcelain (as they are more prone to chipping) [45,46]. As noted above, veneering composites are often used in Japan, and one theory is that this trend is driven by a conceptual assumption that veneering composites are softer than porcelain and less likely to fracture from a materials science standpoint. It can be concluded that the ability to repair prostheses directly in the mouth is also a deciding factor.
  More than half of all repair requests for IODs (i.e., complications) (Q13) (Figure 8) involve fracturing of the denture base or denture tooth detachment (53.8% of all repair requests). The questionnaire also revealed that reconstruction of occlusion because of wear or attrition of denture teeth (24.1%) is a frequent issue leading to laboratory orders. While the literature includes reports of frequent IOD-related prosthetic complications such as attachment-related compromised retention, detachment or fracturing of denture teeth, relining, and attachment damage [25,28,29], this survey showed a somewhat different trend. It can be inferred that these results differ from actual complication trends because they constitute responses to cases sent to labs as repair requests, and because the survey targeted dental technicians. The causes of this phenomenon can be found in responses to other questions as described above. In short, the questionnaire suggested the possibility that inadequate awareness of prosthetics is making IOD complications in Japan more complex, with issues including the comparatively frequent use of resin bases, problems with implant location and orientation, and inadequate consideration of occlusion by dentists.
  Finally, technicians gave voice to the several requests for dentists, who are their customers, as a result of their daily experiences accomplishing implant laboratory procedures (Q14) (Figure 9). These included asking dentists to use suitable implant location and orientation (31.8%), to allow technicians to participate and consult with technicians from the treatment planning stage (28.3%), to improve consideration of soft tissue as well as its condition (21.8%), and to add more in-depth knowledge of prosthesis and occlusal design (14.5%). As observed, implant location and orientation issues in particular not only complicate technical work, but may also cause a variety of complications after the initiation of loading. For cases involving a broad range of implant prostheses and occlusal reconstruction, if not all cases, the dental technicians should be a part of the team from the treatment planning stage to enable restoration-driven implant treatment in the true sense of the term. At the same time, a dentist with an extensive understanding of prosthodontics should play the leading role in treatment of such cases. This survey succeeded in identifying prosthetic problems by examining implant prosthetic complications from the dental technician’s perspective. As stated in the description of the survey’s purpose, it is hoped that dentists make use of this report to reaffirm prosthetic concepts and awareness so that there is achievement of predictable implant prosthetic treatment.

## Conclusions

This survey served to clarify the current status of implant prosthodontics, issues, and considerations in their fabrication, and the status of prosthetic complications and preventive initiatives, all from a laboratory perspective.

1. Concerning implant treatment, it was concluded that dentists either play the leading role or work in collaboration with technicians, including in the formulation of treatment direction and that a team approach has been achieved to a certain extent.
2. This survey identified the problems that technicians address on a frequent basis in the fabrication of prostheses (these should be noted by dentists), including implant location and angulation, impression and bite registration precision, and occlusal considerations.
3. Concerning prevention of veneer fractures, it was also concluded that the best approach consists of metal occlusal (including a metal backing for the distal-most area) and coping designs.
4. The results of this survey suggest that, to fabricate prostheses with a high level of predictability, functional utility, and aesthetic satisfaction, it is necessary to reaffirm the importance of dentists increasing their prosthetic knowledge and working together with dental technicians to develop comprehensive treatment plans, design prostheses, and accomplish occlusal reconstruction.
